# Hemolysis, icterus and lipemia interfere with the determination of two oxidative stress biomarkers in canine serum

**DOI:** 10.1186/s12917-023-03740-y

**Published:** 2023-09-23

**Authors:** B. Perez-Montero, M. L. Fermin-Rodriguez, G. Miro, L. de Juan, F. Cruz-Lopez

**Affiliations:** 1 Clinical Pathology Service, Veterinary Teaching Hospital, Complutense University, Madrid, Spain; 2 Animal Medicine and Surgery Department, Faculty of Veterinary Medicine, Complutense University, Madrid, Spain; 3 Animal Health Department, Faculty of Veterinary Medicine, Complutense University, Madrid, Spain; 4 VISAVET Health Surveillance Centre, Complutense University, Madrid, Spain

**Keywords:** Thiobarbituric acid reactive substances, Total antioxidant status, Jaundice, Interference

## Abstract

**Background:**

Oxidative stress has been proven to play a role in numerous human and canine diseases. Among the biomarkers of oxidative stress, Thiobarbituric Acid Reactive Substances (TBARS) and Total Antioxidant Status (TAS) are two of the most widely used. Preanalytical factors are crucial for obtaining accurate results in these assays. Hemolysis, icterus and lipemia (HIL) are common sources of preanalytical errors in the laboratory; however, limited information is available regarding the considerations for canine specimens. Therefore, the objective of this study was to evaluate the potential interferences of HIL in the determination of TBARS and TAS in canine serum.

**Methods:**

Solutions of pooled canine serum samples were prepared by adding increasing concentrations of hemolysate, bilirubin and a synthetic lipid emulsion. TBARS and TAS were determined, and biases from the control value caused by the interfering substances were calculated.

**Results:**

Hemolysis, icterus and lipemia induced significant interferences on TBARS and TAS, albeit to varying degrees depending on the specific biomarker and interfering substance. TBARS appeared to be more susceptible to interferences in this study. Slight hemolysis, moderate icterus and slight lipemia caused notable deviations in TBARS values, surpassing the acceptable threshold for interference. TAS assay was also affected by HIL, although to a lesser extent compared to TBARS. Significant biases from TAS control value were observed when icterus was moderate, and when hemolysis and lipemia were more pronounced.

**Conclusions:**

In light of our results, we conclude that hemolyzed, icteric and lipemic specimens are not suitable for TBARS and TAS determination in canine serum. Our findings hold considerable practical utility, as a simple visual inspection would be sufficient for identifying and excluding such specimens.

**Supplementary Information:**

The online version contains supplementary material available at 10.1186/s12917-023-03740-y.

## Background

Oxidative stress (OS) is currently described as an imbalance between oxidants and antioxidants, favoring the oxidants, which can disrupt redox signaling and cause molecular damage [1, 2]. This imbalance involves the generation of free radicals and reactive species, specifically reactive oxygen species (ROS). Excessive oxidative challenge has been shown to damage biomolecules and contribute to the development and progression of various diseases [2, 3]. In dogs, oxidative stress has been implicated in conditions such as cardiovascular [4, 5], respiratory [6], hematological [7–9], gastrointestinal [10, 11], renal [12], infectious and parasitic diseases [13–15], as well as cancer [16, 17].

Numerous biomarkers of OS have been identified [18], which reflect either oxidative damage to biomolecules or the body’s antioxidant defenses. Lipid peroxidation can be assessed by measuring specific metabolites such as malondialdehyde (MDA), which is one of the most widely used biomarkers of OS [19]. Circulating MDA can be measured using different techniques, with one of the most commonly used being the Thiobarbituric Acid Reactive Substances (TBARS) assay [20–23]. Another approach to evaluating OS is by assessing the overall antioxidant capacity of a given sample. This can be estimated using different tests, including the 2,2′-azinobis (3- ethylbenzthiazolin-6-sulfonic acid) (ABTS) test, also called Total Antioxidant Status (TAS) assay, which is one of the most extensively employed colorimetric methods in both human and canine species [24–26].

Analytical and preanalytical factors are critical in the determination of these biomarkers [19, 22]. With regards to TBARS, it has been noted that blood sampling and storage conditions may induce artefactual formation of MDA [27]. Additionally, the presence of other compounds containing reactive carbonyl groups in the sample can affect the specificity of TBARS by interfering with the assay through their reaction with thiobarbituric acid (TBA) [19, 22, 27]. In the determination of TAS, analytical and preanalytical variables also play a role. There are different versions of the ABTS-based TAS assay [24, 26, 28, 29], employing varying reagents, measurement wavelengths, and antioxidant molecules that contribute to the overall result [24]. Consequently, the molecules present in the sample have different effects on the assay results.

Hemolysis, icterus and lipemia (HIL) are common sources of erroneous results among clinical laboratories [30–32]. They are considered preanalytical factors that induce endogenous interference, potentially leading to altered assay results [32]. Hemolysis occurs when erythrocytes are disrupted, releasing their intracellular components, particularly hemoglobin. Although it can occur in vivo, it significantly interferes with laboratory results when it happens during or after sample collection [30, 31, 33]. It is considered one of the major causes of unsuitable specimens in clinical laboratories [32–35]. Icterus is caused by increased bilirubin concentrations, typically resulting from liver and biliary duct disease, as well as in vivo hemolysis [30]. Lipemia arises from high levels of lipoprotein particles, such as chylomicrons and very-low density lipoproteins (VLDL), leading to sample turbidity [30, 31]. While recent food intake is the most common cause, lipemia can also be associated with pathological conditions such as metabolic and nutritional disorders, and certain medications, among others [30, 36]. HIL interference can occur through spectral, chemical, dilutional and additive mechanisms, depending on peak absorbance wavelengths, potential cross-reactions between various molecules, and the release of intraerythrocytic substances. Consequently, the nature of HIL interference depends on the specific analytical method employed [30, 32].

The literature on the interference of HIL on TBARS and TAS assays is scarce, both in human [24, 34] and veterinary medicine [26, 37]. Two studies have assessed the influence of hemolysis and lipemia on TBARS and TAS in canine serum [26, 37], but their results show some discrepancies and they do not evaluate the interference caused by icterus. Hence, the aim of our study was to evaluate the potential interference resulting from increasing concentrations of hemoglobin, bilirubin, and lipids in canine serum on TBARS and TAS. Considering the significant evidence linking OS to various pathological conditions in dogs, understanding the impact of preanalytical factors on these assays is of utmost importance.

## Results

### Effect of hemolysis

**Fig. 1:**
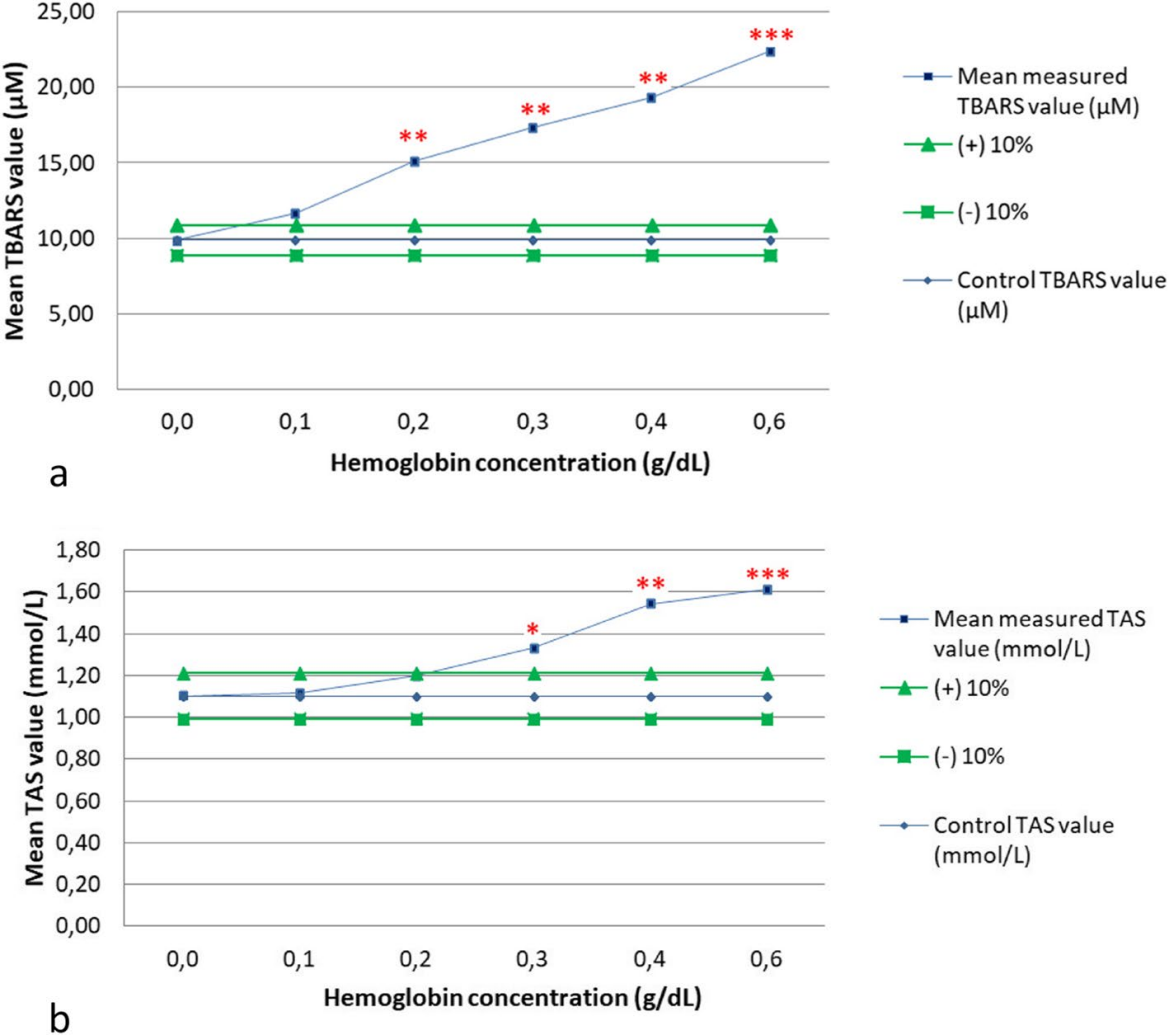
**a**, **b** Interpherographs showing the effects of hemolysis on Thiobarbituric Acid Reactive Substances (TBARS) (Fig. 1a) and Total Antioxidant Status (TAS) (Fig. 1b) assays in canine serum. *p-*value significance: *p < 0.05; **p < 0.01; ***p < 0.001. Graphs show spiked solutions with increasing concentrations of hemoglobin on the x-axes, mean concentrations of the analytes on the y-axes, *p* values and 10% threshold of interference. These graphics are a modification of those proposed by Glick, et al*.* [38, 39]

The interferences of hemolysis on TBARS and TAS are listed in Table 1 and shown in interferographs (Fig. 1a and b). The control serum (not spiked) had a TBARS value of 9.86 µM, and even slight hemolysis resulted in an observed deviation of 18.31%, exceeding the 10% bias. Mean TBARS values increased further with the addition of hemolysate, up to 22.36 µM (126.76% bias) at a hemoglobin (Hb) concentration of 0.6 g/dL. The control serum showed a TAS value of 1.10 mmol/L. Slight and moderate hemolysis had no significant effect. However, in the presence of marked and extreme hemolysis, the value of TAS was significantly overestimated (over 20.70% bias), leading to results as high as 1.61 mmol/L.

**Table 1:** Effects of hemolysis on Thiobarbituric Acid Reactive Substances (TBARS) and Total Antioxidant Status (TAS) assays in canine serum. *p-*value significance: *p < 0.05; **p < 0.01; ***p < 0.001. Hb: Hemoglobin. SD: Standard deviation

**Spiked solutions**	**Hb (g/dL)**	**Hemolysis degree (visual estimation)**	**Mean TBARS value (µM)**	**SD**	***p*-value**		**Observed % bias**	**Mean TAS value (mmol/L)**	**SD**	***p*-value**		**Observed % bias**
1	0.0	Absent	**9.86**	0.39				**1.10**	0.03			
2	0.1	Slight	**11.67**	0.98	0.068812		18.31	**1.12**	0.04	0.342757		1.20
3	0.2	Moderate	**15.14**	0.39	0.002789	**	53.52	**1.20**	0.06	0.077423		8.71
4	0.3	Marked	**17.36**	0.39	0.001388	**	76.06	**1.33**	0.04	0.011852	*	20.70
5	0.4	Extreme	**19.31**	0.79	0.002152	**	95.77	**1.54**	0.01	0.001286	**	39.87
6	0.6	Extreme	**22.36**	0.00	0.000251	***	126.76	**1.61**	0.01	0.000799	***	46.30

### Effect of icterus

**Fig. 2:**
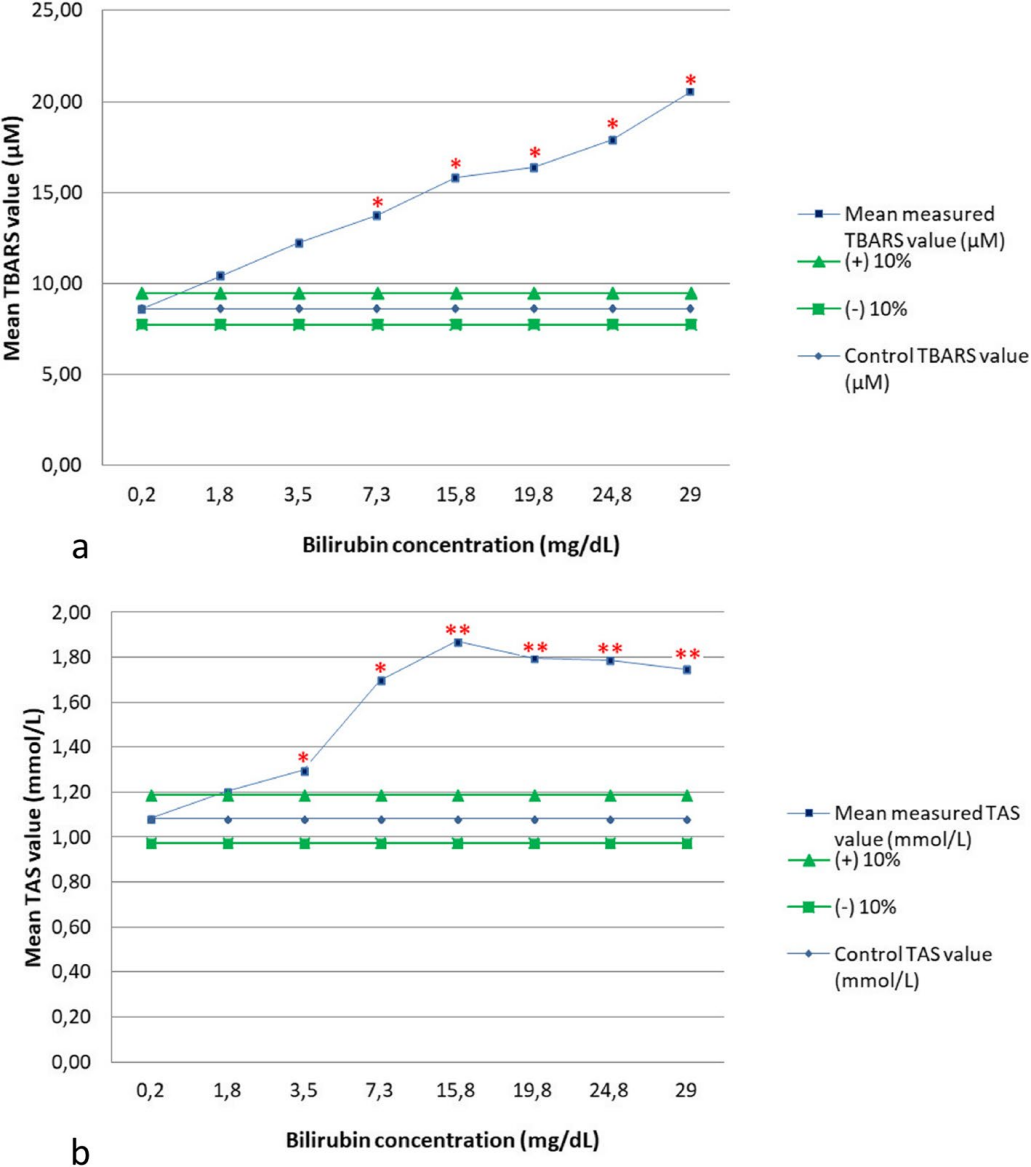
**a**, **b** Interpherographs showing the effects of icterus on Thiobarbituric Acid Reactive Substances (TBARS) (Fig. 2a) and Total Antioxidant Status (TAS) (Fig. 2b) assays in canine serum. *p-*value significance: *p < 0.05; **p < 0.01. Graphs show spiked solutions with increasing concentrations of bilirubin on the x-axes, mean concentrations of the analytes on the y-axes, *p* values and 10% threshold of interference. These graphics are a modification of those proposed by Glick, et al*.* [38, 39]

Hyperbilirubinemia resulted in significant overestimation of both TBARS and TAS (Tables 2, 3, and Fig. 2a and b). In the first experiment, the initial non-spiked serum with a bilirubin concentration of 0.2 mg/dL, showed TBARS and TAS values of 8.61 µM and 1.08 mmol/L, respectively. The first spiked sample had a bilirubin concentration of 1.8 mg/dL, and both TBARS and TAS were significantly interfered with values of 10.42 µM and 1.20 mmol/L, respectively. Therefore, a second experiment was performed (Table 3) to define a more accurate threshold. In this case, a bilirubin concentration of 1.1 mg/dL (moderate icterus) resulted in an overestimation of both TBARS and TAS beyond the 10% bias limit. In the case of TAS, this increase continued until a bilirubin concentration of 15.8 mg/dL, when it reached a plateau at approximately 1.8 mmol/L.

**Table 2:** Effects of icterus on Thiobarbituric Acid Reactive Substances (TBARS) and Total Antioxidant Status (TAS) assays in canine serum. First experiment. *p-*value significance: *p < 0.05; **p < 0.01. SD: Standard deviation

**Spiked solutions**	**Bilirubin (mg/dL)**	**Icterus degree (visual estimation)**	**Mean TBARS value (µM)**	**SD**	***p*-value**		**Observed % bias**	**Mean TAS value (mmol/L)**	**SD**	***p*-value**		**Observed % bias**
1	0.2	Absent	**8.61**	1.77				**1.08**	0.10			
2	1.8	Marked	**10.42**	0.00	0.142298		20.97	**1.20**	0.02	0.111456		11.23
3	3.5	Extreme	**12.22**	0.59	0.055531		41.94	**1.30**	0.01	0.046033	*	19.93
4	7.3	Extreme	**13.75**	0.39	0.028432	*	59.68	**1.70**	0.08	0.010158	*	57.13
5	15.8	Extreme	**15.83**	1.37	0.022462	*	83.87	**1.87**	0.01	0.003958	**	73.07
6	19.8	Extreme	**16.39**	0.20	0.012578	*	90.32	**1.79**	0.00	0.004748	**	66.06
7	24.8	Extreme	**17.92**	1.18	0.01256	*	108.06	**1.79**	0.03	0.005175	**	65.46
8	29.0	Extreme	**20.56**	4.12	0.031914	*	138.71	**1.75**	0.03	0.005801	**	61.71

**Table 3:** Effects of icterus on Thiobarbituric Acid Reactive Substances (TBARS) and Total Antioxidant Status (TAS) assays in canine serum. Second experiment. *p-*value significance: *p < 0.05. SD: Standard deviation

Spiked solutions	Bilirubin (mg/dL)	Icterus degree (visual estimation)	Mean TBARS value (µM)	SD	*p*-value	Observed % bias	Mean TAS value (mmol/L)	SD	*p*-value	Observed % bias
1	< 0.1	Absent	**7.36**	0.79			**0.96**	0.02		
2	0.3	Absent	**7.50**	0.59	0.43206	1.89	**1.01**	0.03	0.114242	5.06
3	1.1	Moderate	**8.61**	0.59	0.107774	16.98	**1.07**	0.00	0.014118*	10.98

### Effect of lipemia

**Fig. 3:**
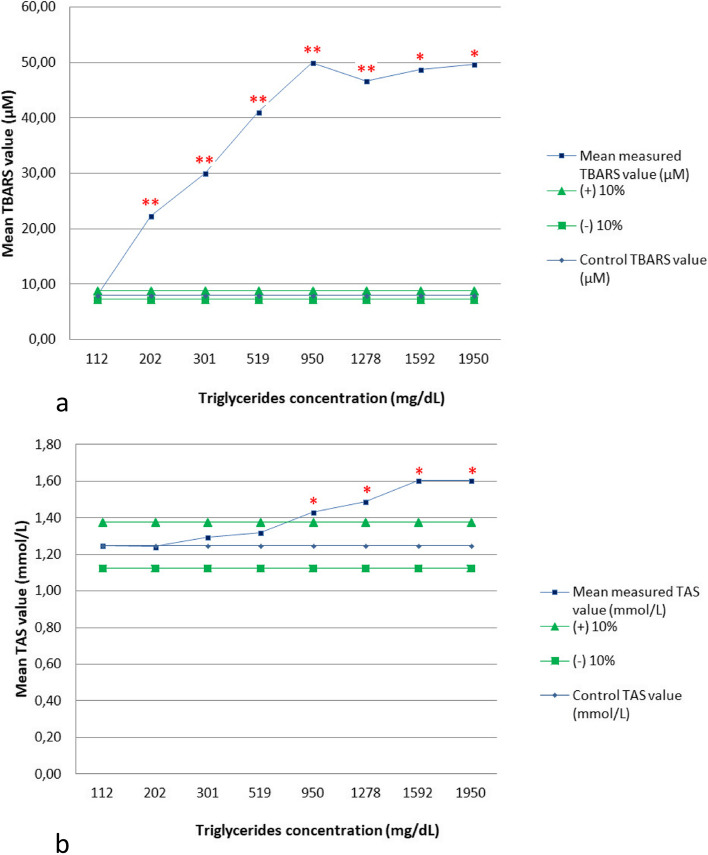
**a**, **b** Interpherographs showing the effects of lipemia on Thiobarbituric Acid Reactive Substances (TBARS) (Fig. 3a) and Total Antioxidant Status (TAS) (Fig. 3b) assays in canine serum. *p-*value significance: *p < 0.05; **p < 0.01. Graphs show spiked solutions with increasing concentrations of triglycerides on the x-axes, mean concentrations of the analytes on the y-axes, *p* values and 10% threshold of interference. These graphics are a modification of those proposed by Glick, et al*.* [38, 39]

The addition of the lipid emulsion had different effects on TBARS and TAS, as shown in Tables 4, 5 and Fig. 3a and b. While the control serum showed a TBARS value of 8.06 µM, the first spiked solution [with a triglycerides (TG) concentration of 202 mg/dL] showed a TBARS value of 22.36 µM (177.59% bias). The mean TBARS value sharply increased to 50 µM (520.69% bias) when lipemia was marked (950 mg/dL of TG) and then remained stable despite the addition of higher lipid concentrations (Table 4). To further investigate the effect of lower concentrations of TG, a second experiment was performed, but did not exceed the 10% bias limit (Table 5). The TAS value of the control serum was 1.25 mmol/L, and the addition of lipids up to 519 mg/dL had no significant effect. Only marked lipemia with TG of 950 mg/dL caused a 14.43% bias in the TAS value. When lipemia was extreme (1950 mg/dL) the bias increased further to 28.26%.

**Table 4:** Effects of lipemia on Thiobarbituric Acid Reactive Substances (TBARS) and Total Antioxidant Status (TAS) assays in canine serum. First experiment. *p-*value significance: *p < 0.05; **p < 0.01. TG: Triglycerides. SD: Standard deviation

**Spiked solutions**	**TG (mg/dL)**	**Lipemia degree (visual estimation)**	**Mean TBARS value (µM)**	**SD**	***p*-value**		**Observed % bias**	**Mean TAS value (mmol/L)**	**SD**	***p*-value**		**Observed % bias**
1	112	Absent	**8.06**	1.37				**1.25**	0.05			
2	202	Slight	**22.36**	1.57	0.005249	**	177.59	**1.24**	0.02	0.458761		-0.59
3	301	Moderate	**30.00**	1.37	0.001952	**	272.41	**1.29**	0.04	0.220384		3.53
4	519	Marked	**41.11**	4.52	0.005028	**	410.34	**1.32**	0.02	0.133766		5.40
5	950	Marked	**50.00**	7.27	0.007601	**	520.69	**1.43**	0.00	0.024335	*	14.43
6	1278	Extreme	**46.67**	5.30	0.004959	**	479.31	**1.49**	0.01	0.014093	*	19.04
7	1592	Extreme	**48.75**	12.96	0.023853	*	505.17	**1.60**	0.07	0.017397	*	28.36
8	1950	Extreme	**49.72**	12.37	0.020934	*	517.24	**1.60**	0.06	0.01379	*	28.26

**Table 5:** Effects of lipemia on Thiobarbituric Acid Reactive Substances (TBARS) assay in canine serum. Second experiment. TG: Triglycerides. SD: Standard deviation

Spiked solutions	TG (mg/dL)	Lipemia degree (visual estimation)	Mean TBARS value (µM)	SD	*p*-value	Observed % bias
1	86	Absent	**7.92**	0.39		
2	111	Absent	**8.06**	0.20	0.346256	1.75
3	161	Slight	**8.47**	0.39	0.146454	7.02

### Intra-assay coefficients of variation (CV)

The intra-assay coefficients of variation for this protocol (16 samples) were 7.48% for TBARS and 4.04% for TAS and thus did not exceed the 10% bias threshold established for the interference limit.

## Discussion

Preanalytical interferences have been noted with some OS biomarkers, but, to our knowledge, this is the first study to assess the interference of hemolysis, icterus and lipemia on TBARS and TAS in canine serum.

In the study of analytical interferences, the establishment of cut-off thresholds is of great importance. Several approaches have been proposed, but there is no clear consensus on the criteria for setting these thresholds [35]. The Clinical and Laboratory Standards Institute (CLSI) recommends the use of an analyte-specific approach that accounts for bias, imprecision and physiological variability of each biomarker [30, 40]. However, this approach was not applicable in our study because the intra-individual biological variation coefficients for canine TBARS and TAS were unknown. On the other hand, manufacturers often use a 10% bias from the control value to set the interference threshold [30, 32, 38, 39]. This approach has also been used in previous interference studies [31, 41–43]. In the present study, we used both a 10% threshold and a *t*-test. We ultimately chose the 10% threshold because it yielded more restrictive limits than the *t*-test in all our experiments.

### TBARS

Determination of MDA by the TBARS assay appeared to be the most susceptible method to interferences in our study. Spectral interference could be a possible explanation. Oxygenated and deoxygenated hemoglobin show different absorbances between 400 and 600 nm, with a peak around 415 nm [30, 32, 44]. Bilirubin absorbs light between 340 and 540 nm, with a peak around 450–460 nm [30, 32, 44]. Lipids, on the other hand, absorb light in a broad range of wavelengths across the visual spectrum (300–700 nm) [30]. TBARS was determined at 540 nm in this study according to the manufacturer’s protocol. Therefore, HIL may have interfered with the spectrophotometric measurement of TBARS.

We found that even slight hemolysis (0.1 g/dL Hb concentration) induces significant interference with TBARS. It is known that hemolysis can contribute to the artificial formation of MDA [19, 34]. Interference of hemolysis with MDA determination by gas chromatography tandem mass spectrometry (GC–MS/MS) has been reported [34]. The reason would be ex vivo peroxidation of free and esterified polyunsaturated fatty acids (PUFAs), especially arachidonic acid. Free hemoglobin could potentially catalyze the formation of MDA from these PUFAs [19, 34]. In addition, it has been suggested that certain intraerythrocytic components may induce MDA production when released into the plasma in hemolytic samples because of the lower antioxidant properties of plasma compared with the intraerythrocytic compartment [34]. As with other analytes [30], it is possible that multiple mechanisms contribute to the hemolysis interference in this assay.

In our study, we found a remarkable bias in the TBARS value in the moderately icteric sample (1.1 mg/dL bilirubin), which increased further upon addition of bilirubin. In addition to spectral interference, analytical interference due to cross-reactivity may also explain the results. The specificity of TBARS can be affected by the presence of other compounds (carbohydrates, oxidized PUFAs and amino acids) that can react with thiobarbituric acid (TBA) [19, 27]. This cross-reaction has already been noted for bilirubin and biliverdin [23, 27]. It is conceivable that it may have overestimated the final value in this case.

The effect of lipemia was even more dramatic. Even slight lipemia (202 mg/dL TG) resulted in significant interference, which increased to 520.69% of bias when lipemia was marked (950 mg/dL). The other lipids spiked in the sample may have cross-reacted with TBA, which could be supported by the fact that TBARS measures lipid peroxidation but is not specific for MDA. It has been reported that TBA can react with other aldehydes [20, 22, 45] and thus possibly with other lipids. This is in contrast to a previous report in which no correlation was found between lipemia and TBARS levels in canine serum [37]. However, we used a commercial TBARS kit, whereas a different TBARS protocol was used in that study, and special caution should be used when comparing TBARS results from different laboratories [22].

### TAS

TAS assay was also affected by HIL, but to a lesser extent than TBARS. In the TAS assay, spectral interference seems less likely to explain the interferences due to HIL for two reasons. First, TAS is determined at 660 nm in this commercial kit. This wavelength is outside the reported absorbance ranges of hemoglobin and bilirubin [30, 32, 44]. It matches only certain lipids absorbance, in the edge of their absorbance range. Second, the assay consists of two absorbance measurements, before and after incubation, and takes into account only the increase in absorbance. Therefore, a possible influence of turbidity seems unlikely.

In contrast to a previous report that found a negative interference of hemolysis but used a different method [26], we observed a significant increase in the TAS value upon addition of hemolysate. However, this resulted in a significant bias only when hemolysis was marked (0.3 g/dL Hb). We used a hemolysate instead of adding only hemoglobin to test the effects of all intraerythrocytic components. Red blood cells (RBCs) are equipped with a strong antioxidant network consisting of enzymes (glutathione peroxidase-1, catalase and peroxiredoxin-2), reduced thiols and vitamins [46–48]. Hemoglobin also possesses antioxidant properties thanks to certain amino acid residues [47]. We suggest that the release of intraerythrocytic components may have exerted antioxidant effects that overestimated the TAS value.

Hyperbilirubinemia had a major effect on the TAS value. Bilirubin concentrations equal or higher than 1.1 mg/dL induced a significant percentage of positive bias, which further increased. Bilirubin acts as an endogenous antioxidant in serum [49, 50], scavenging various reactive species that contribute to OS, especially hydrogen peroxide (H_2_O_2_). This ability to react with H_2_O_2_ is also a known mechanism of interference on several analytical methods [30, 44]. We consider it conceivable that the increase in TAS, which we obtained when bilirubin was added, was due to this mechanism. Moreover, some authors have already recognized the ability of the TAS assay to detect the antioxidant properties of bilirubin [24, 29].

Lipemia moderately affected the TAS assay. Slight and moderate lipemia did not greatly affect the TAS value. It was significantly affected only when lipemia was marked (950 mg/dL TG or more). Two previous studies examined the interference of TG concentrations up to 500 mg/dL on TAS value in dogs but did not evaluate the effects of higher concentrations [26, 37]. One of these studies found no effect on TAS up to the aforementioned TG concentration, which is consistent with our results [26]. Bonatto et al*.* found an increase in TAS value when a commercial lipid emulsion was spiked, whereas they found a decrease in TAS in dogs with postprandial lipemia [37]. It has been noted that synthetic lipid emulsions cannot perfectly mimic the lipemic state in vivo because of differences in serum lipid composition [51]. However, there is a lack of materials to better simulate lipemia, as opposed to the case of hemolysis and icterus.

## Conclusion

In view of our results, hemolyzed, icteric, and lipemic samples would not be suitable for the determination of TBARS and TAS in canine serum. Our results appear to be of considerable practical utility because the interferences found in this study occurred in hemolysis, icterus, and lipemia degrees that are detectable by simple visual inspection. Therefore, we consider it advisable to discard hemolyzed, icteric and lipemic samples for the determination of TBARS and TAS in canine serum.

## Methods

### Study design

This study followed the guidelines provided by the Clinical and Laboratory Standards Institute (CLSI) [40], the American Society for Veterinary Clinical Pathology (ASVCP) [52], and the Spanish Society of Laboratory Medicine (SEQC) for interference testing [44]. It also incorporated modifications from the recommendations of Glick et al*.* [38]. In summary, OS biomarkers were measured in canine pooled serum samples with increasing concentrations of interferents (HIL), and the biases due to the interferents were then calculated. To assess the inherent biases of the techniques, intra-assay coefficients of variation were also determined. Pooled serum was obtained from multiple samples from healthy and sick dogs submitted to the Clinical Pathology Service of the Veterinary Teaching Hospital (Complutense University of Madrid) for routine analysis.

### Preparation of hemolysate and hemolyzed serum pools

To obtain the hemolysate, a canine blood sample that was submitted to the laboratory for routine analysis was used. Briefly, 2 mL of canine blood in a lithium heparine tube were centrifuged for 10 min at 1200 *g.* Plasma was separated for routine biochemistry analysis. Then, 4 mL of physiological saline solution was added to the remaining centrifuged sample, and the tube was homogenized. This process of centrifugation and wash in saline was repeated 3 times. The washed red blood cells (RBCs) were lysed with 1 mL of distilled water and frozen at -20ºC for at least 12 h. The sample was thawed, vigorously vortexed and centrifuged again to remove cell debris. The hemoglobin concentration of the hemolysate was measured as 10,3 g/dL using Drabkin’s method, as recommended [44]. The generated lysate was stored at -20ºC until further experiments.

To prepare the spiked solutions, 10 mL of non-hemolyzed pooled canine sera were aliquoted into two tubes, each containing 4.5 mL of serum. One tube received 250 µL of distilled water (non-spiked serum, control), and the other received 250 µL of hemolysate (spiked serum). Six solutions were prepared with decreasing concentration of non-spiked serum and increasing concentrations of spiked serum to reach a final volume of 500 µL in each solution (see Supplementary material Table 1). The Hb concentration of each solution was measured as previously described. The solutions were intended to correspond to increasing degrees of hemolysis, ranging from absent (0.0 g/dL) to extreme hemolysis (0.4 g/dL onwards).

### Preparation of icteric serum pools

To investigate the interference of icterus, 30 mg of bilirubin (Sigma-Aldrich, USA) were dissolved in 5 mL of 0.1 mol/L NaOH. Similar to the hemolyzed serum pools, 10 mL of non-icteric pooled canine serum were divided in two tubes: non-spiked serum (4.5 mL of serum and 250 µL of distilled water) and spiked serum (4.5 mL of serum and 250 µL of bilirubin solution). Non-spiked serum was mixed with increasing volumes of spiked serum to obtaining a final volume of 500 µL in eight solutions (see Supplementary material Table 2a). The bilirubin concentration of each solution was measured using a biochemistry analyzer (IDEXX Catalyst One®, USA) and ranged from absent (0.2 mg/dL) to extreme icterus (29 mg/dL). A second experiment was conducted to examine the effect of lower concentrations of bilirubin. Three additional solutions were prepared to yield bilirubin concentrations of < 0.1, 0.3, and 1.1 mg/dL, representing slight icterus (see Supplementary material Table 2b).

### Preparation of lipemic serum pools

A commercial lipid emulsion (Intralipid® 20%, Fresenius Kabi, Germany) was used to test the effect of lipemia. Again, non-lipemic pooled canine serum was divided into two tubes: non-spiked serum (4.5 mL of serum and 250 µL of distilled water) and spiked serum (4.5 mL of serum and 250 µL of Intralipid®). Eight solutions were prepared to yield increasing lipid concentrations, as previously described (see Supplementary material Table 3a). The final TG concentrations were measured using a biochemistry analyzer (IDEXX Catalyst One®, USA) and ranged from 112 to 1950 mg/dL (representing extreme lipemia). Additionally, further solutions were prepared to achieve lower TG concentrations of 86, 111 and 161 mg/dL, representing slight lipemia (see Supplementary material Table 3b).

### Oxidative stress biomarkers

Lipid peroxidation was assessed by means of a TBARS assay kit (Cayman Chemicals, USA), previously used in canine serum and plasma samples [53, 54]. The method is based on the reaction between MDA and TBA under acidic conditions and high temperatures. The MDA-TBA adduct is measured spectrophotometrically at 540 nm and the results are expressed in MDA concentration (µM).

Antioxidant capacity was evaluated using an assay based on the reaction between ABTS, a peroxidase (metmyoglobin), and hydrogen peroxide, which produces a blue-green color. This color change is determined by measuring the absorbance value at 660 nm using the TAS-liquid stable kit (Fortress Diagnostics Limited, UK). The results are expressed in mmol Trolox Equivalent/L. The method was previously validated in canine serum samples [26].

Both biomarkers were measured in duplicate in each solution of the samples and mean measured value as well as standard deviation (SD) were calculated.

### Intra-assay CV

The imprecision of the techniques was evaluated using the intra-assay CV. Sixteen replicates of TBARS and TAS were determined using a canine pooled serum. The intra-assay CV for each technique was calculated as the SD divided by the mean value of the analyzed replicates [16], and then multiplied by 100 to obtain the percentage (CV = (SD/mean)*100).

## Statistical analysis

Results from the non-spiked control serum and the interferent-containing samples were compared using Student’s *t*-test for independent means. A *p* value of < 0.05 was considered statistically significant. Additionally, the bias due to the interferent was expressed as percentage of bias, following the formula: Bias (%) = 100 × (measured value − control value)/control value. The measured value represented the apparent analyte concentration in the interferent-containing sample, and the control value was the analyte concentration in the non-spiked sample. Significant interference was defined as a change of the analyte value in the spiked sample exceeding 10% of the non-spiked sample value [30–32, 38, 39, 41–44]. Both the *t-*test and the 10% bias limit were considered when examining the results, and the more restrictive criterion was chosen. Interferographs were constructed based on the proposed methodology by Glick, et al*.* [38, 39].

### Supplementary Information

**Additional file 1.**

## Data Availability

The data and materials are available from the corresponding author upon reasonable request.

## Abbreviations

ASVCP: American Society for Veterinary Clinical Pathology
CLSI: Clinical and Laboratory Standards Institute
CV: Coefficient of variation
GC–MS/MS: Gas chromatography tandem mass spectrometry
H_2_O_2_: Hydrogen peroxide
Hb: Hemoglobin
HIL: Hemolysis, icterus and lipemia
MDA: Malondialdehyde
OS: Oxidative stress
PUFAs: Polyunsaturated fatty acids
ROS: Reactive oxygen species
SD: Standard deviation
SEQC: Spanish Society of Laboratory Medicine
TAS: Total Antioxidant Status
TBA: Thiobarbituric Acid
TBARS: Thiobarbituric Acid Reactive Substances
TG: Triglycerides
VLDL: Very-low density lipoproteins
