# Supplementary material for: Hemolysis, icterus and lipemia interfere with the determination of two oxidative stress biomarkers in canine serum
Source: BMC Vet Res. 2023 Sep 23;19:172. doi: 10.1186/s12917-023-03740-y (PMC10517482; doi:10.1186/s12917-023-03740-y)
Supplement: Supplementary file 1 — Additional file 1. [file 12917_2023_3740_MOESM1_ESM.docx]

| Spiked solutions | Non-spiked serum (µL) | Spiked serum (µL) | Final  volume (µL) |
| --- | --- | --- | --- |
| 1 | 500 | 0 | 500 |
| 2 | 450 | 50 | 500 |
| 3 | 400 | 100 | 500 |
| 4 | 300 | 200 | 500 |
| 5 | 100 | 400 | 500 |
| 6 | 0 | 500 | 500 |

**Supplementary material, Table 1: Hemolysis.** Volumes of non-spiked and spiked serum added to generate spiked solutions, with increasing concentrations of the interferent (hemoglobin).

| Spiked solutions | Non-spiked serum (µL) | Spiked serum (µL) | Final  volume (µL) |
| --- | --- | --- | --- |
| 1 | 500 | 0 | 500 |
| 2 | 475 | 25 | 500 |
| 3 | 450 | 50 | 500 |
| 4 | 400 | 100 | 500 |
| 5 | 300 | 200 | 500 |
| 6 | 200 | 300 | 500 |
| 7 | 100 | 400 | 500 |
| 8 | 0 | 500 | 500 |

**Supplementary material, Table 2a: Icterus (first experiment).** Volumes of non-spiked and spiked serum added to generate spiked solutions, with increasing concentrations of the interferent (bilirubin).

| Spiked solutions | Non-spiked serum (µL) | Spiked serum (µL) | Final  volume (µL) |
| --- | --- | --- | --- |
| 1 | 500 | 0 | 500 |
| 2 | 495 | 5 | 500 |
| 3 | 487.5 | 12.5 | 500 |

**Supplementary material, Table 2b: Icterus (second experiment).** Volumes of non-spiked and spiked serum added to generate spiked solutions, with increasing concentrations of the interferent (bilirubin).

| Spiked solutions | Non-spiked serum (µL) | Spiked serum (µL) | Final  volume (µL) |
| --- | --- | --- | --- |
| 1 | 500 | 0 | 500 |
| 2 | 475 | 25 | 500 |
| 3 | 450 | 50 | 500 |
| 4 | 400 | 100 | 500 |
| 5 | 300 | 200 | 500 |
| 6 | 200 | 300 | 500 |
| 7 | 100 | 400 | 500 |
| 8 | 0 | 500 | 500 |

**Supplementary material, Table 3a: Lipemia (first experiment).** Volumes of non-spiked and spiked serum added to generate spiked solutions, with increasing concentrations of the interferent (triglycerides).

| Spiked solutions | Non-spiked serum (µL) | Spiked serum (µL) | Final  volume (µL) |
| --- | --- | --- | --- |
| 1 | 500 | 0 | 500 |
| 2 | 495 | 5 | 500 |
| 3 | 487.5 | 12.5 | 500 |

**Supplementary material, Table 3b: Lipemia (second experiment).** Volumes of non-spiked and spiked serum added to generate spiked solutions, with increasing concentrations of the interferent (triglycerides).
